# Memoranda on Anatomy, Surgery, and Physiology

**Published:** 1849-01

**Authors:** 


### ARTICLE X.

*Memoranda on Anatomy, Surgery, and Physiology; forming a Pocket Companion for the young Surgeon or the Student, preparing for Examination.* By Mark Noble Bower, Surgeon. Corrected and enlarged. By an American Physician. pp. 320, 18mo. New York, Samuel S. & William Wood, 1848. (From the publishers, and for sale by J. Keene & Bro. Chicago.)

The title given above sufficiently indicates the object of the small book before us.

We like the plan of combining instruction upon anatomy, physiology, and surgery, in the same volume, as it gives the student a better opportunity of observing the practical bearing upon other branches of the knowledge acquired during his investigations upon any one in particular. We cheerfully recommend the work to young practitioners and students, as being well adapted to recall to mind knowledge already acquired from more elaborate works. H.

